# Flexible Anti-Metal RFID Tag Antenna Based on High-Conductivity Graphene Assembly Film

**DOI:** 10.3390/s21041513

**Published:** 2021-02-22

**Authors:** Bohan Zhang, Cheng Zhang, Yuchao Wang, Zhe Wang, Chengguo Liu, Daping He, Zhi P. Wu

**Affiliations:** 1 Hubei Engineering Research Center of RF-Microwave Technology and Application, Wuhan University of Technology, Wuhan 430070, China; zhangbohan@whut.edu.cn (B.Z.); czhang2020@whut.edu.cn (C.Z.); yuchao9629@whut.edu.cn (Y.W.); wangzhe0614@whut.edu.cn (Z.W.); hedaping@whut.edu.cn (D.H.); z.p.wu@whut.edu.cn (Z.P.W.); 2 School of Information Engineering, Wuhan University of Technology, Wuhan 430070, China; 3 State Key Laboratory of Silicate Materials for Architectures, Wuhan University of Technology, Wuhan 430070, China

**Keywords:** graphene assembly film, RFID tag antenna, high conductivity, flexible, anti-metal

## Abstract

We propose a flexible anti-metal radio frequency identification (RFID) tag antenna based on a high-conductivity graphene assembly film (HCGAF). The HCGAF has a conductivity of 1.82 × 10^6^ S m^−1^, a sheet resistance of 25 mΩ and a thickness of 22 μm. The HCGAF is endowed with high conductivity comparable to metal materials and superb flexibility, which is suitable for making antennas for microwave frequencies. Through proper structural design, parameter optimization, semiautomatic manufacturing and experimental measurements, an HCGAF antenna could realize a realized gain of –7.3 dBi and a radiation efficiency of 80%, and the tag could achieve a 6.4 m read range at 915 MHz on a 20 × 20 cm^2^ flat copper plate. In the meantime, by utilizing flexible polyethylene (PE) foam, good conformality was obtained. The read ranges of the tags attached to curved copper plates with different bending radii were measured, as well as those of those attached to several daily objects. All the results demonstrate the excellent performance of the design, which is highly favorable for practical RFID anti-metal applications.

## 1. Introduction

As an essential information collection method for the perception layer in the Internet of Things (IoT), radio frequency identification (RFID) technology in the ultra-high-frequency (UHF) band, due to its passive, wireless and uniquely identifying attributes, has been widely used in logistics tracking, smart retail, health care and asset management [1,2,3,4]. A RFID system consists of a reader, a tag and middleware. Various forms of tags can satisfy different application scenarios. The tag is usually composed of an antenna, chip and substrate. The antenna, as a critical component for receiving and transmitting power, has been extensively studied to achieve remote tracking and intelligent monitoring [5,6,7]. However, when the RFID tag is placed on a metallic surface, the radiation performance of the ordinary dipole antenna still deteriorates due to the image current on the metallic surface [8]. Numerous antenna design solutions have been proposed to overcome this problem, such as an antenna based on an electromagnetic bandgap (EBG) [9,10] or artificial magnetic conductor (AMC) structure [11,12], a patch-style antenna including a planar inverted-F antenna (PIFA) [13,14], and a microstrip-slot antenna [15,16]. These existing designs can favor anti-metal application in many common application scenarios. Still, several shortcomings have been proved such as the high profile, the usage of rigid substrates and the complex structure, restricting its conformal application on curved metallic surfaces [17,18,19,20].

To date, researchers have made a great effort to realize a flexible anti-metal RFID tag. For example, a low-profile patch antenna constructed from a low-loss polymer flexible substrate and thin metal (used for radiation and ground) are proposed to meet the flexibility demand. The substrates include but are not limited to polyvinyl chloride (PVC), polypropylene (PP), polyethylene (PE) and polydimethylsiloxane (PDMS) [21,22,23,24]. However, most of the existing flexible anti-metal tag antennas are made of aluminum foil (~10 μm), the performance of which dramatically deteriorates due to oxidation, corrosion and bending. Once failed, these tags are difficult to degrade and cause a large amount of electronic waste that can induce secondary damage to the natural environment [25,26]. Besides, the chemical etching process terribly pollutes the environment. Therefore, it is attractive to search for a kind of environmentally friendly, chemically stable and mechanically flexible material instead of traditional metals to make RFID tag antennas. Of course, a high conductivity is always necessary.

Several attempts have been made to replace traditional metallic materials by utilizing the physical and chemical properties of carbon materials, such as carbon nanotubes [27,28] and graphene-based composites [29,30,31,32,33,34]. Nevertheless, the unsatisfactory conductivity of carbon nanotubes causes poor radiation performance. Graphene inks [33] and graphene papers [34], as graphene-based composites, have conductivities of 3.7 × 10^4^ and 4.2 × 10^5^ S m^−1^, respectively. Although the tag antenna based on these materials demonstrates the flexibility of carbon-based materials, the radiation performance of the tag antenna is still insufficient for realizing anti-metal applications due to the relatively low conductivities. Fortunately, a multilayered graphene film, presented by our team, has been used in filter and energy harvester applications due to its high conductivity and flexibility [35,36], providing a promising way to tackle the problems previously mentioned.

For the first time, to the best of our knowledge, this paper proposes a flexible anti-metal RFID tag antenna based on a high-conductivity (up to 1.82 × 10^6^ S m^−1^) graphene assembly film (HCGAF). The tag consists of an HCGAF patch antenna, flexible PE foam substrate and chip, which has been designed, optimized and tested. The realized gain, radiation efficiency, radiation pattern and read range performance of the tag on a 20 × 20 cm^2^ flat copper plate were measured and found to be consistent with the simulation results. In particular, the read range tests of the tags were conducted on copper plates with different sizes and bending radii, as well as on metallic surfaces of six daily objects, to prove that our design can be used in conformal applications. The paper is arranged as follows. Section 2 provides a description of the preparation and characterization of the HCGAF, and explains the mechanism of its high conductivity and flexibility from the material microstructure. Section 3 describes the design and measurement of the HCGAF antenna, which provided support for the subsequent tag manufacturing and measurements. Section 4 shows the experimental results illustrating the flexible anti-metal performance of the tag based on the HCGAF antenna. Section 5 draws conclusions.

## 2. Material Preparation and Characterization

**Figure 1:**
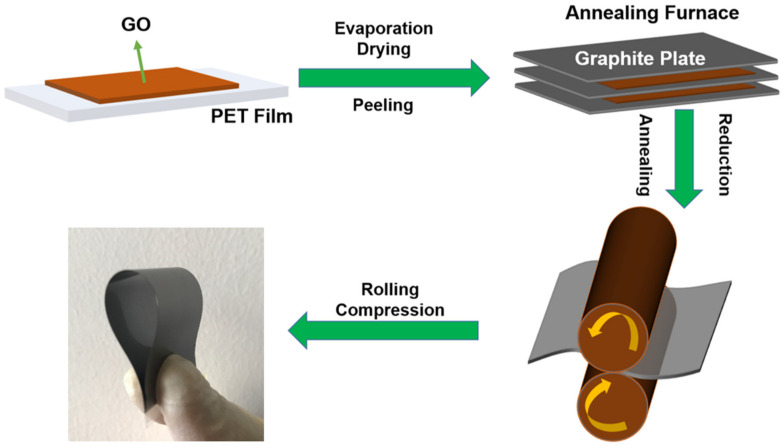
Schematic diagram of high-conductivity graphene assembly film (HCGAF) fabrication process.

As is shown in Figure 1, the fabrication of the HCGAF included the following steps: the self-assembly of a graphene oxide (GO) suspension by evaporation at room temperature, high-temperature heat treatment, and static calendering. The GO suspension was diluted with ultrapure water to a concentration of 15 mg mL^−1^ in a square vessel. After mechanical stirring at room temperature, the diluted GO suspension was evaporated to obtain the GO film. The GO film was annealed in an Ar gas flow furnace at the high temperatures of 1300 and 3000 °C for 2 and 1 h, respectively, achieving the reduction and graphitization of pre-HCGAF. The pre-HCGAF was attached to the Polyethylene Terephthalate (PET) substrate by static calendering with a pressure of 100 MPa for 0.5 h at room temperature, and then, the HCGAF was formed; a photograph of the HCGAF under a bending state is shown in Figure 1. To ensure a material with an ideal structure of graphene layers and high conductivity, the following characterization of the HCGAF was carried out.

**Figure 2:**
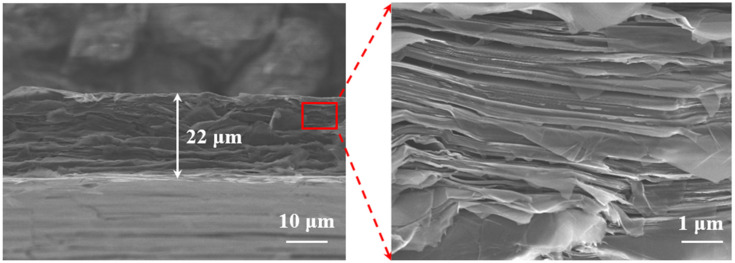
The cross-sectional and the highly magnified SEM images of the HCGAF.

First, the material structure was inspected with the JSM-7100F scanning electron microscope (SEM). In Figure 2, the cross-sectional and the highly magnified SEM images of the HCGAF show that the film is based on orderly stacked graphene layers with a thickness of 22 μm. The skin depth, ***δ_s_***, is related to the frequency and is an intrinsic property of a conductive material. It is defined as the thickness of the material that ensures the effective flow of electrical currents. When the field in the conductor is transmitted a distance of the skin depth, its amplitude decays to 1/e. The ***δ_s_*** of the HCGAF was 12 μm at 900 MHz; the HCGAF’s thickness had enough space for electrical current flow.

**Figure 3:**
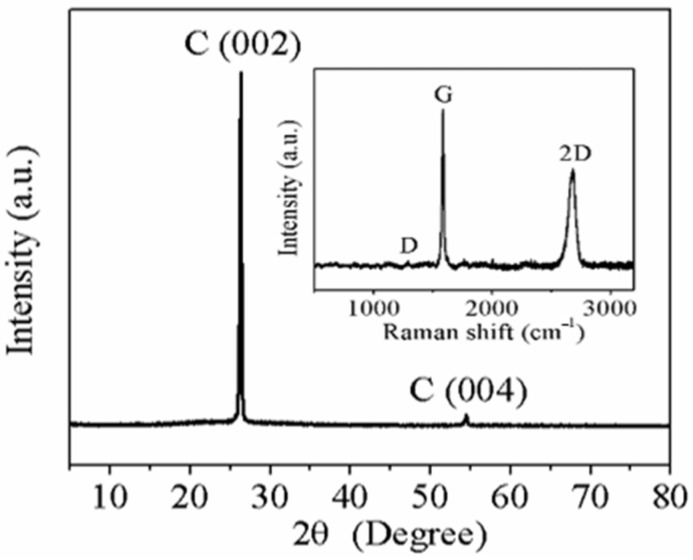
XRD pattern of HCGAF (inset: Raman spectroscopy of HCGAF).

Second, XRD patterns were collected using X-ray D/MAX-RB instruments with Cu Ka radiation, and Raman spectroscopy was performed using a Renishaw Raman Spectrometer. The sharp diffraction peak of the (002) plane in the X-ray diffraction (XRD) pattern was located at 2θ = 26.5° (Figure 3), indicating an interlayer spacing of 0.336 nm. The unobvious D band (1329 cm^−1^) and strong 2D band (2684 cm^−1^) in the Raman spectroscopy (inset of Figure 3) indicate a highly graphitized structure [37,38]. Additionally, due to the π–π interactions of 2D nanosheets in the graphene layer, the 2D nanosheets can easily slide between each other along the plane and maintain great adhesion power, leading to the remarkable mechanical and electrical stability of the HCGAF upon bending.

**Figure 4:**
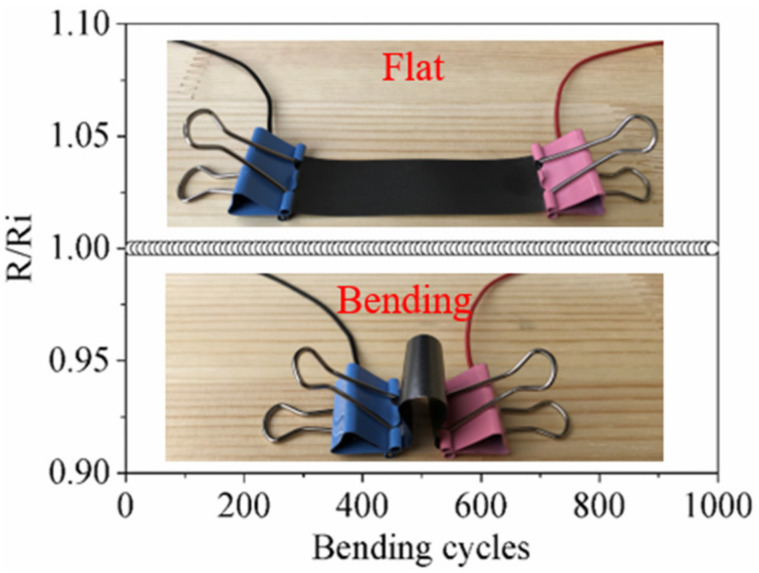
Mechanical flexibility test of the HCGAF strip.

Then, in order to demonstrate the excellent mechanical flexibility, the rectangular HCGAF strip was bent from flat to nearly 90° for repetitive cycles, and the two ends of the HCGAF strip were connected with a multimeter to measure the transient electrical resistance, as shown in Figure 4. Even after a thousand bending cycles, the HCGAF strip without fracture still kept a constant relative resistance that was normalized to the initial ultra-low resistance value, while a metallic strip will break after about 200 bending cycles.

**Figure 5:**
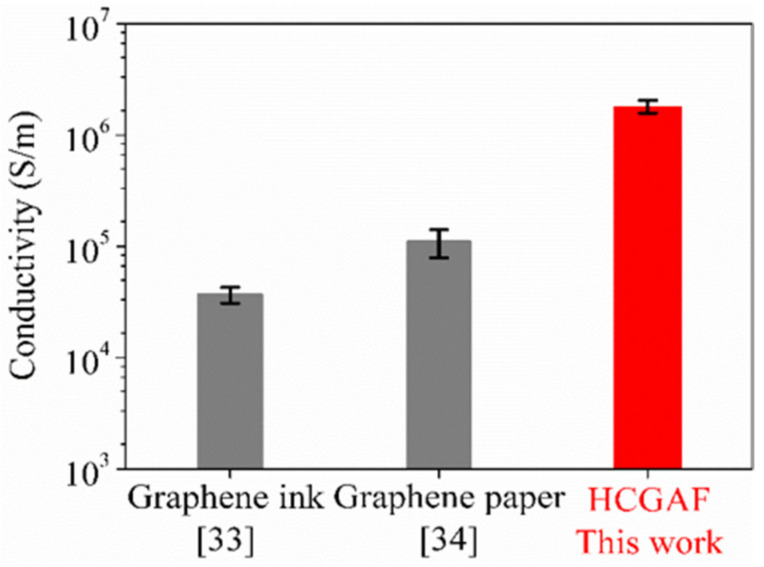
Conductivity comparison of three materials.

Meanwhile, the conductivity, ***σ***, was measured by the Four-Point Probes Resistivity Measurement System (RTS-9). As shown in Figure 5, the HCGAF had a conductivity of 1.82 × 10^6^ S m^−1^, which is at least one order of magnitude higher than the conductivity of graphene-based films reported previously. The sheet resistance, ***R_t_***, was also measured; the lower the sheet resistance of the material, the faster the electrical currents flow on its surface. As an index to judge the conductivity of materials with, the sheet resistance can be calculated from the conductivity and thickness. The sheet resistance of the HCGAF was 25 mΩ, which is the lowest value for graphene-based films to date. In terms of graphene ink, polymer-stabilizing agents (such as cellulose acetate butyrate) are often added into the inks so that the graphene flakes can be uniformly dispersed and the printing performance is improved. The insulating polymer-stabilizing agents attached to the surface of the graphene sheets will inevitably lead to a sharp increase in the resistance of the graphene and a decrease in the conductivity [33]. Meanwhile, graphene papers are prepared by the compression of the powder of graphene nanoplatelets under some pressure at room temperature. Without a high-temperature heat-treatment process, the self-healing of the sp^2^ structure in graphene papers cannot be realized, and the contact resistance of the internal structure is large, which leads to the resistance still being large [34]. The measurement results for the conductivity and sheet resistance show that the HCGAF is suitable as an antenna for receiving and transmitting electromagnetic waves of microwave frequency.

## 3. HCGAF Antenna Design and Measurements

### 3.1. Complex Impedance Conjugate Matching Theory

In the RFID system, the tag antenna receives power from the reader and transmits the power to the chip. When the power is sufficient to activate the chip, the modulation and backscattering process of the chip is triggered, and the backscattered power is transmitted back to the reader through the tag antenna [6]. In the tag antenna design, the antenna’s power reflection coefficient, ***S***_11_, and the power transfer coefficient, ***τ***, describe the power transfer ratio from the antenna to the chip, which can be calculated by the following equations:

(1) $$S_{11}=\frac{Z_{c}-Z_{a}^{*}}{Z_{c}+Z_{a}}$$

(2) $$\tau =1-{\left|S_{11}\right|}^{2}=\frac{4R_{a}R_{c}}{|Z_{a}+Z_{c}{|}^{2}}0\leq \tau \leq 1$$

where ***Z_a_*** = ***R_a_*** + *j**X_a_*** and ***Z_c_*** = ***R_c_*** + *j**X_c_*** are the input complex impedance of the antenna and the characteristic complex impedance of the chip, respectively. According to the conjugate matching principle, the power delivered to the chip reaches a maximum when ***Z_a_*** = Z_c_^*^, i.e., ***τ*** = 1. However, in the tag production, due to the manufacturing tolerance, the complete conjugate matching between the antenna and chip cannot be realized in the whole UHF band. Therefore, the conjugate matching value of the center frequency is usually chosen for antenna design [39]. In our design and experiments, the chips of the Impinj Monza R6 series were used [40]. Based on the R6 datasheet, the chip is modeled by using a resistor of 1.2 kΩ and a capacitor of 1.44 pF (containing a typical capacitance of 0.21 pF due to adhesive and antenna mount parasitics) in parallel configuration, which results in a complex impedance of 12 − j119 Ω (capacitive) at the resonant frequency of 920 MHz. Hence, the antenna complex impedance should be designed to be 12 + j119 Ω (inductive). Since the R6 chip has two differential RF input pads to connect the antenna, the two antenna ports are designed to be kept on a horizontal line with an interval of 0.15 mm.

### 3.2. Design of HCGAF Antenna

**Figure 6:**
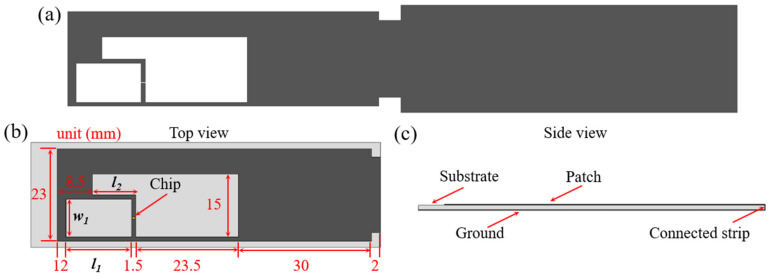
The structure of the proposed antenna: (**a**) tile view of the antenna without wrapping the substrate; (**b**) top view and (**c**) side view of the antenna wrapped around the substrate.

The structure of the proposed anti-metal RFID tag antenna made of the HCGAF is shown in Figure 6a. The antenna consists of a radiation patch, a ground and a connected strip. The rectangular slot and the inverted-L-shaped slot on the patch can not only increase the electrical length to achieve the miniaturization of the antenna, but also provide the inductive reactance to facilitate conjugate matching with the capacitive reactance of the chip [41]. The patch is connected to the ground by a short circuit strip, and then, the antenna wraps around the flexible polyethylene (PE) foam substrate (the dielectric constant ***ε_r_*** = 1.22) with a thickness of 1 mm to form a current loop without adopting metallic via holes. The corresponding structural parameters can be observed in Figure 6b,c.

**Figure 7:**
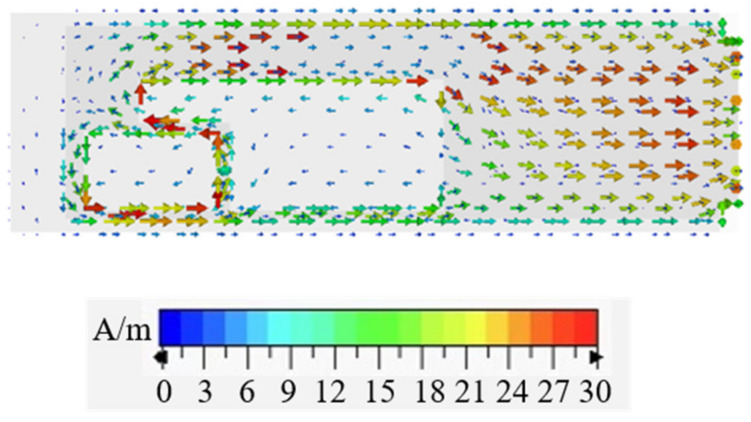
Simulated surface electrical current distribution of the antenna at 920 MHz.

The parameter optimization of the anti-metal tag antenna was carried out in the CST Microwave Studio Suite. When conducting the simulation, the tag was placed at the center of a 20 × 20 cm^2^ flat copper plate. Before performing a parameter scan, the simulated surface electrical current distribution of the antenna at 920 MHz was as provided in Figure 7. The arrows represent the surface electrical current flow path, and the amplitudes of the surface electrical currents are distinguished by colors. It was found that high current densities were concentrated around the rectangular slot and the insertion part of the inverted-L-shaped slot, indicating that the complex impedance and resonance frequency of the antenna can be effectively affected by changing the rectangular slot length, ***l_1_***; the rectangular slot width, ***w_1_***; and the inverted-L-shaped slot insertion length, ***l_2_***.

**Figure 8:**
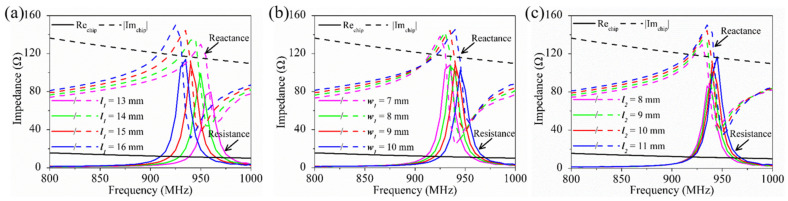
Simulated complex impedance of the antenna with different parameter values: (**a**) ***l_1_***, (**b**) ***w_1_*** and (**c**) ***l_2_***.

To reveal the influence rule, the simulated resistance (solid color lines) and reactance (dashed color lines) of the antenna complex impedance versus frequency for different ***l_1_*** are shown in Figure 8a. The solid black line and the dashed black line represent the resistance and reactance of the chip’s calculated conjugate complex impedance, respectively. As ***l_1_*** increased from 13 to 16 mm, it can be observed that the resistance of the antenna increased from 4 to 30 Ω at the resonant frequency of 920 MHz, and the reactance increased from 99 to 140 Ω simultaneously. The conjugate complex impedance curves of the chip intersect with the curves of ***l_1_*** = 15 mm at 920 MHz, where the complex impedance of the antenna was 12 + j 120 Ω, and thus, the maximum power transmission from the antenna to the chip could be achieved.

Similarly, the effects of different ***w_1_*** and ***l_2_*** on the simulated complex impedance of the antenna are also analyzed in Figure 8b,c, respectively. The resistance of the antenna decreased from 29 to 8.5 Ω at 920 MHz when ***w_1_*** increased from 7 to 10 mm, and at the same time, the reactance decreased from 130 to 117 Ω. However, the opposite phenomena can be observed when ***l_2_*** increased from 8 to 11 mm; the resistance decreased but the reactance increased. According to the previous analysis, ***l_1_*** is convenient to roughly modulate the antenna complex impedance with, while ***w_1_*** and ***l_2_*** are more suitable for finetuning. Therefore, by synergistically adjusting ***l_1_***, ***w_1_*** and ***l_2_***, the final optimized values of ***l_1_***, ***w_1_*** and ***l_2_*** were determined to be 15, 9 and 10 mm, respectively.

### 3.3. Performance Measurements of HCGAF Antenna

**Figure 9:**
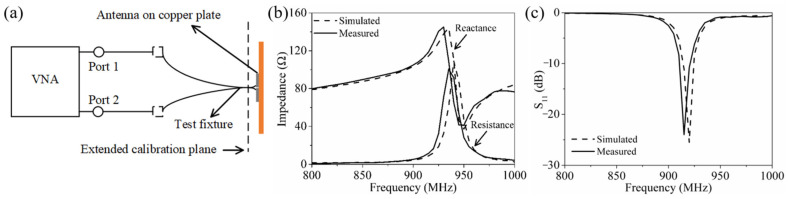
Antenna performance measurements: (**a**) schematic diagram; simulated and measured (**b**) complex impedance and (**c**) reflection coefficient of the antenna with optimized parameters.

To validate the feasibility of the simulation, the optimized HCGAF antennas were fabricated and measured. The prepared initial HCGAF was placed on the PET substrate; the HCGAF antennas were patterned accurately by the LPKF laser machine with a programmed path. After the redundant HCGAF was peeled off from the PET substrate, the HCGAF antennas were formed. The antenna complex impedance can be measured by the two-port complex impedance measurement method [42], as shown in Figure 9a. The test fixture consisted of two semirigid coaxial cables. At one end of the test fixture, the two outer conductors of the coaxial cables were soldered together, and the two inner cores were extended and connected to the two ports of the antenna with a conductive epoxy (Circuit works CW2400). At the other end, the test fixture was connected to a vector network analyzer (VNA, Agilent E5072A) using Sub-Miniature version A (SMA) connectors and cables. After extending the calibration plane to the end of the test fixture connecting the antenna ports and measuring the ***S*** parameters of the two-port network, the complex impedance of the antenna ***Z_a_*** can be obtained by the following formula:

(3) $$Z_{a}=\frac{2Z_{0}(1-S_{11}S_{22}+S_{12}S_{21}-S_{12}-S_{21})}{(1-S_{11})(1-S_{22})-S_{21}S_{12}}$$

where ***Z***_0_ = 50 Ω. ***S***_11_, ***S***_12_, ***S***_21_ and ***S***_22_ are four measured ***S*** parameters of two-port networks. Figure 9b illustrates the simulated and measured complex impedance of the antenna with optimized parameters over a frequency range of 800–1000 MHz; the measured complex impedance was in good agreement with the simulated one. The frequency at which the measured complex impedance matched the conjugate complex impedance of the chip was 915 MHz, and the simulated one was 920 MHz. Figure 9c shows the simulated and measured reflection coefficient of the antenna with optimized parameters. The measured reflection coefficient reached a minimum of –24 dB at 915 MHz, which is 1.5 dB higher than the simulated value. Still, there was a 5 MHz deviation between the two resonance frequencies. Note that the slight discrepancy between the two results is due to the mild complex impedance mismatch caused by soldering the test fixture to the antenna port.

## 4. Tag Measurements and Conformal Applications

The performance of the tag was evaluated on a 20 × 20 cm^2^ flat copper plate, including the realized gain, radiation efficiency, read range and radiation pattern, and the effect of different sizes of flat copper plate on the read range of the tag was also studied. Then, the read range of the tag was tested on curved copper plates with different radii and daily objects, and the conformal applicability of the tag was verified. These aspects are described in Section 4.1 to Section 4.3.

### 4.1. Tag Measurements on Flat Copper Plates

**Figure 10:**
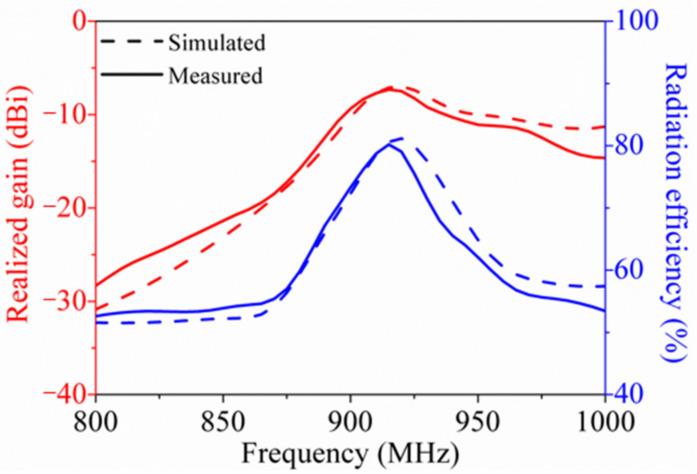
Simulated and measured realized gain and radiation efficiency of the tag.

In the process of tag fabrication, the chips were connected to the HCGAF antennas with the anisotropic conductive heat-curing adhesive Delo AC265. The adhesive dispensing and flip chip bonding were performed by a semiautomatic packaging machine. Although complex impedance conjugate matching between an antenna and a chip has been well designed, manufacturing tolerances are inevitable when the chip is bonded to the antenna. In order to further evaluate the overall performance of the tag, the realized gain was simulated (***G_r, sim_***) and measured (***G_r, mea_***) on the basis of the following equations:

(4) $$G_{r,sim}=G_{t}\tau ,$$

(5) $$G_{r,mea}=\frac{P_{c}}{P_{th}L_{fwd}},$$

where ***G_t_*** is the gain of the tag, ***P_c_*** is the chip sensitivity, ***P_th_*** is the measured threshold power, and ***L_fwd_*** is the forward path loss from the reader output port to the tag input port. Among these parameters, ***G_t_*** and ***τ*** could be acquired by simulation, and ***P_c_*** was provided by the chip manufacturer (for the R6 chip, ***P_c_*** = −20 dBm), while ***P_th_*** and ***L_fwd_*** needed to be measured during calibration. The realized gain and radiation efficiency measurements of the tag were performed using the VNA, Diamond Engineering Antenna Measurement Studio and a rotary table in an anechoic chamber. As shown in Figure 10, the measured peak realized gain could reach –7.3 dBi at 915 MHz, which is 0.7 dBi lower than that of the simulated one, despite a little frequency offset (–6.6 dBi at 920 MHz), since the transmission path loss and manufacturing tolerance were not considered during the simulations. Besides, the radiation efficiency, as another vital evaluation index for the antenna, was also determined. It is evident that the radiation efficiency reached 80% at 915 MHz, fitting well with the simulated ones.

**Figure 11:**
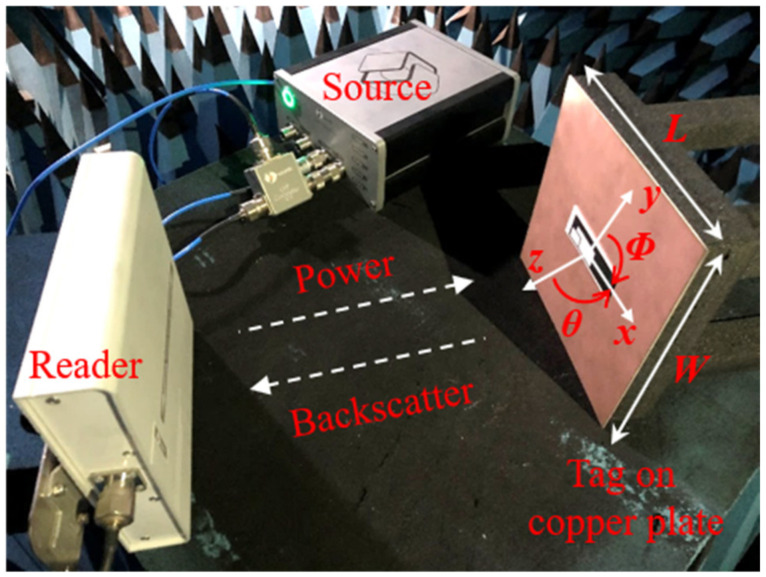
The tag under test in the anechoic chamber.

As the most intuitive and essential performance parameter of the tag, the simulated (***R_sim_***) and measured (***R_mea_***) read range of the tag could be obtained using the following equations:

(6) $$R_{sim}=\frac{\lambda }{4\pi }\sqrt{\frac{P_{reader}G_{reader}G_{t}}{P_{t}}}=\frac{\lambda }{4\pi }\sqrt{\frac{EIRP\cdot G_{r,sim}}{P_{c}}},$$

(7) $$R_{mea}=\frac{\lambda }{4\pi }\sqrt{\frac{EIRP\cdot G_{r,mea}}{P_{c}}}=\frac{\lambda }{4\pi }\sqrt{\frac{EIRP}{P_{th}L_{fwd}}},$$

where ***λ*** is the antenna wavelength at the resonant frequency, ***P_t_*** is the tag received power and ***P_t_*** = ***P_c_τ***. ***P_reader_*** and ***G_reader_*** are the radiated power and the realized gain of the reader, respectively. ***EIRP*** = ***P_reader_G_reader_*** is the equivalent isotropically radiated power of the reader; the default value of ***EIRP*** is 3.28 W in China and Europe; for countries that allow a 4.00 W ***EIRP***, the measurement result needs to be increased by 11%. By substituting Equations (4) and (5) into Equations (6) and (7), ***R_sim_*** could be calculated according to the Friss formula, and ***R_mea_*** was implemented using the Voyantic Tagformance Pro RFID measurement system in the anechoic chamber, as shown in Figure 11. During the test, the tag was placed on the center of a copper plate with a length of ***L*** and a width of ***W***, the radiation direction of the linearly polarized antenna in the reader was along the boresight (***φ*** = 0°, ***θ*** = 0°) of the tag, the transmission power range of the reader was 0 to 27 dBm, and the receiver of the reader had a sensitivity of −75 dBm. The transmission power was increased by the source in steps of 0.1 dBm until the tag was activated, at which point the transmission power value was ***P_th_***. Then, a valid response (backscatter) to the inquiry command for the Electronic Product Code (EPC) Type 1 Gen 2 protocol was received from the tag by the reader. The frequency was swept from 800 to 1000 MHz in steps of 1 MHz.

**Figure 12:**
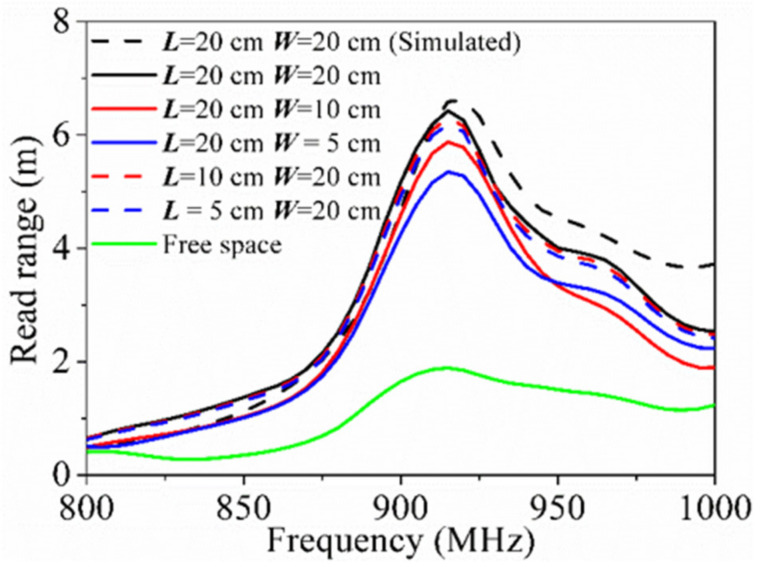
Simulated read range of the tag on a 20 × 20 cm^2^ copper plate, measured read ranges of the tag on copper plates of different sizes and measured read range of the tag in free space.

In Figure 12, the simulated and measured read ranges of the tag on a copper plate with ***L*** = 20 cm and ***W*** = 20 cm are represented by a black dashed line and black solid line, respectively. It is obvious that the measured read range (6.4 m at 915 MHz) of the tag was almost consistent with the simulated one (6.6 m at 920 MHz), despite the frequency deviation and little degradation of the performance. Besides, we investigated the effect of the ground on the read range by changing its size. It should be noted that the measured read range remained stable when ***L*** decreased from 20 to 5 cm but ***W*** = 20 cm was fixed (red and blue dashed lines). On the contrary, if we changed ***W*** but held ***L***, the test results gradually decreased from 6.4 to 5.3 m as ***W*** was reduced (red and blue solid lines). Even like this, it still maintained high-level performance. In addition, the read range of the tag dramatically deteriorated when getting rid of the metallic plate.

**Figure 13:**
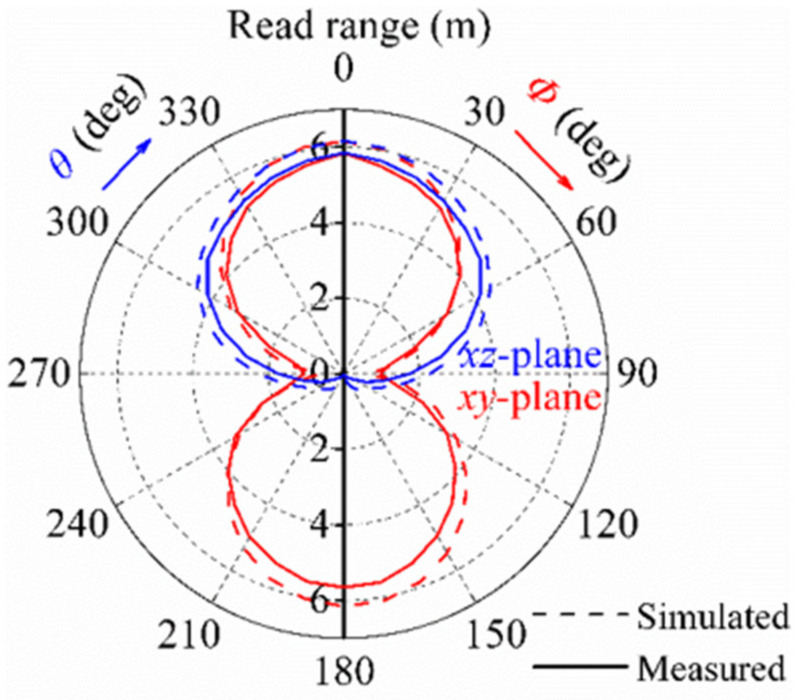
Simulated and measured radiation patterns in terms of the read range of the tag on a 20 × 20 cm^2^ flat copper plate at 915 MHz in ***xy*** plane and the ***xz*** plane.

To comprehensively reveal the performance of our design backed by a 20 × 20 cm^2^ flat copper plate, the measured and simulated two-dimensional (2D) radiation patterns in terms of the read range at 915 MHz in the ***xy*** plane and ***xz*** plane are presented in Figure 13. Concerning the ***xy*** plane, the measured read range exceeded 4 m in the angular ranges of |***φ***| ≤ 50° and |***φ***| ≥ 130°. Unlike for the ***xy*** plane, due to the shielding effect, the measured read range across the ***xz*** plane was almost zero, when |***θ***| was larger than 90 degrees.

### 4.2. Tag Measurements on Curved Copper Plates

**Figure 14:**
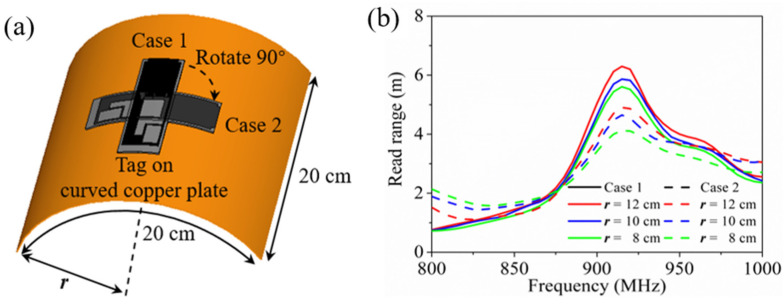
Tag measurements on curved copper plate: (**a**) schematic diagram; (**b**) measured read ranges of the tag on curved copper plates with the same area but different bending radii.

For practical anti-metal applications, RFID tags are often used in curved surfaces and objects. Hence, the conformal performance of the proposed HCGAF-based tag was evaluated as follows. As shown in Figure 14a, our design was attached to a regular curved copper plate with the same area (both the side length and arc length were 20 cm) but a varying bending radius ***r*** to measure its read range. For case 1, the tag was bent along its short edge, and for case 2, the tag was rotated by 90 degrees; that is to say, the tag was bent along its long edge.

The measured read ranges of the tag on curved copper plates with the same area but different bending radii are shown in Figure 14b; as the ***r*** of the curved copper plate decreased from 12 to 8 cm, it can be observed that the read range of the tag decreased from 6.2 to 5.5 m at 915 MHz in case 1 (solid lines), and the read range of the tag decreased from 4.9 to 4.1 m at 915 MHz in case 2 (dash lines). The read range always decayed gradually within an acceptable level, which was caused by the deformation of the tag antenna and the reduction of the effective radiation aperture. Note that as a result of the smaller shape changes, the tag in case 1 could acquire a longer read range than that of case 2 in terms of the same ***r***. It is also worth mentioning that there was almost no shift in the resonant frequency of the tag, even though the tag had a certain deformation.

### 4.3. Tag Conformal Application on Daily Metallic Objects

**Figure 15:**
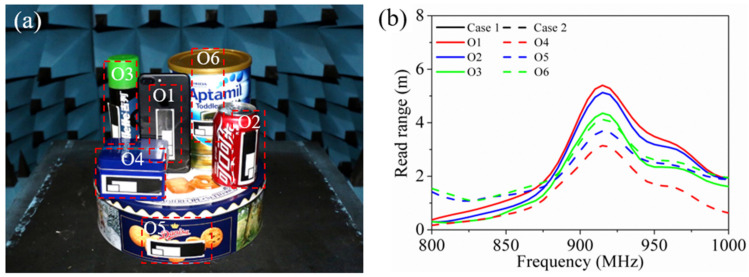
Tag conformal application on daily metallic objects: (**a**) schematic diagram; (**b**) measured read ranges of the tag on the 6 objects.

Moreover, considering daily objects, the tags were attached to six different metallic objects (O1–O6, namely, a mobile phone, Coca-Cola container, shaving foam container, tea container, cookie container and milk powder container), as shown in Figure 15a.

In Figure 15b, the measured read ranges of the tag on the six objects are given, which are also divided into case 1 (O1–O3, solid lines) and case 2 (O4–O6, dashed lines). The tag had a maximum read range of 5.4 m at 915 MHz on the flat surface of O1, of which the surface area and width were the largest. The tag had a minimum read range of 3.1 m at 915 MHz on O4, since the surface area and width of O4 were the smallest. Although the bending radius of O6 was smaller than that of O4 and O5, due to the larger surface area and width, the corresponding tag shared a longer read range. The tags operated on almost the same frequency band, regardless of the sizes and bending radii of the metallic surfaces. Finally, the on-site demonstration was performed by placing the objects on a customized pallet equipped with a near-field UHF reader. As demonstrated in Video S1, when the object was put down or picked up from the pallet, the corresponding picture of the object marked by the tag could be recorded on the display. In addition to the pictures, the text information of the objects can be written into the chip memory, uploaded to the cloud and displayed on the terminal, which is conducive to the traceability of the objects.

## 5. Conclusions

In summary, an HCGAF-based RFID tag was investigated and measured to realize flexible anti-metal performance for conformal applications. Through the reasonable design of the tag antenna, conjugate matching with a chip was achieved to realize perfect power transfer. Instead of employing metallic via holes to connect to ground, a wrapped patch-slot antenna was put forward to obtain a realized gain of –7.3 dBi and a radiation efficiency of 80%, and therefore, our tag can successfully obtain an excellent read range of 6.4 m at 915 MHz on a 20 × 20 cm^2^ flat copper plate. Due to the low profile as well as usage of the HCGAF and the flexible PE foam, the proposed tag shows a good advantage for conformal applications. The read ranges of the tag attached to the regular curved copper plate and more general daily objects were also measured, and the high-level read range at the steady operation frequency prove the flexible feature as previously described. There is no report of using graphene materials as antennas to make anti-metal RFID tags, because the conductivity of graphene materials is much lower than that of the HCGAF [29,30,31,32,33,34]. However, the flexibility of anti-metal tags made of traditional aluminum antennas cannot be compared with that of graphene materials [21,22,23,24]. Hence, the robust performance indicates that our design paves a new avenue for the IoT and other real-life applications.

## Data Availability

Not applicable.

## Supplementary Materials

The following are available online at https://www.mdpi.com/1424-8220/21/4/1513/s1, Video S1: Demonstration of HCGAF-based tag conformal application on daily metallic objects.
